# Detailed Study of Amplitude Nonlinearity in Piezoresistive Force Sensors

**DOI:** 10.3390/s110908836

**Published:** 2011-09-14

**Authors:** Leonel Paredes-Madrid, Luis Emmi, Elena Garcia, Pablo Gonzalez de Santos

**Affiliations:** Centre for Automation and Robotics, UPM-CSIC, Ctra. Campo Real Km 0.2, Madrid 28500, Spain; E-Mails: luis.emmi@car.upm-csic.es (L.E.); elena.garcia@car.upm-csic.es (E.G.); pablo.gonzalez@car.upm-csic.es (P.G.S.)

**Keywords:** piezoresistive, force, sensor, nonlinear, model, tanh, piezocapacitive, FlexiForce

## Abstract

This article upgrades the RC linear model presented for piezoresistive force sensors. Amplitude nonlinearity is found in sensor conductance, and a characteristic equation is formulated for modeling its response under DC-driving voltages below 1 V. The feasibility of such equation is tested on four FlexiForce model A201-100 piezoresistive sensors by varying the sourcing voltage and the applied forces. Since the characteristic equation proves to be valid, a method is presented for obtaining a specific sensitivity in sensor response by calculating the appropriate sourcing voltage and feedback resistor in the driving circuit; this provides plug-and-play capabilities to the device and reduces the start-up time of new applications where piezoresistive devices are to be used. Finally, a method for bypassing the amplitude nonlinearity is presented with the aim of reading sensor capacitance.

## 1. Introduction

Piezoresistive force sensors have demonstrated to be a good solution for applications demanding non-invasive force readings [1–6]. However, the relatively low repeatability and considerable hysteresis of piezoresistive force sensors in comparison with load cells [7] limit the use of such sensors to applications where accuracy is not as important as the size of the sensor involved [8,9].

On the other hand, load cells have demonstrated to be reliable force-measurement devices in many different systems [10,11] demanding high reliability and accuracy [12], e.g., force-control applications [13] and impact measurements [14]. However, because of the relative bulk and weight of load cells as compared to piezoresistive sensors, when a new robot or force-control system is under design, load-cell bulk and weight must be taken into account from the early stages of design.

In certain research fields, such as biomechanics, biomedical engineering and haptics, it is necessary to perform non-invasive force readings. Such readings cannot be carried out using bulky load cells. Whether it is necessary to measure contact force on a knee joint [15,16], measure the grasp force of a human hand [1,4] or accommodate any of many other applications [17–19], a low-profile, light-weight sensor must be used in order to meet the limited space requirements of the task.

Previous work [20,21] has demonstrated that the repeatability of piezoresistive force sensors may be increased by performing capacitance readings under AC sourcing. The method detailed in [20,21] consists in reading sensor conductance and capacitance by applying DC and sine waveforms; thereby it is possible to perform a multivariable estimation of force that dramatically reduces force-estimation errors.

In [20,21] an RC-parallel-electrical model was identified for the FlexiForce A201-100 piezoresistive force sensor (PFS); in addition, a frequency nonlinearity in sensor response when the frequency of the driving signal exceeded a certain value was found and appropriately named “the divergent frequency”. However, we have recently found that the PFS exhibits additional nonlinearities related with the amplitude of the driving signal, and thus the RC model and the corresponding equations must be modified to account for this newly found amplitude nonlinearity.

The A201-100 piezoresistive sensor was chosen for this study because the manufacturer has developed many specific sensors for research [2,22], medical [23] and service applications, and thus the A201-100 is widely used in many different fields. This paper reviews the RC model for the A201-100 force sensor, which is referred to henceforth as “the PFS”. The PFS nonlinearities are studied and different methods for bypassing them are proposed. A generalized method for obtaining a specific sensitivity is also detailed. This involves choosing the proper DC amplitude and feedback resistor in the conditioning circuit, a procedure restricted to sourcing voltages below 1 V. Finally, a method is detailed for bypassing the amplitude nonlinearity under AC sourcing with the aim of performing capacitance readings on the sensor.

## 2. Previous Work on Modelling the Piezoresistive Sensor Model A201-100

**Figure 1.**
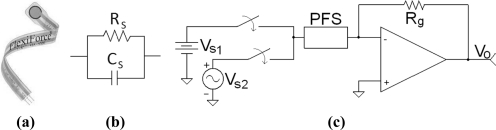
Piezoresistive sensor under study. **(a)** Picture of the PFS. **(b)** Circuit model of the PFS. **(c)** Conditioning circuit for measuring forces in the PFS.

Most research articles regarding the A201-100 sensor present performance comparisons among different piezoresistive devices [7,8] or describe applications developed using the aforementioned sensor [2,3,15]. Only [20,21] present an electrical characterization of the device. In [20,21], a RC-parallel-electrical model [see Figure 1(b)] was identified for the PFS under study [see Figure 1(a)] with a typical driving circuit as shown in Figure 1(c) [24]. When the sine wave, *V_s2_*, is selected as input, the following set of equations from [20] describes sensor response within its linear region of operation. First, a differential equation can be deduced from the circuit depicted in Figure 1(c) with *V_s2_* as input:

(1) $$\frac{V_{s2}}{R_{s}}+C_{s}\frac{{\mathrm{dV}}_{s2}}{\mathrm{dt}}=-\frac{V_{o}}{R_{g}}$$

where *R_s_* and *C_s_* are sensor resistance and capacitance, respectively, from the model in Figure 1(b).

Parameter *R_g_* is the feedback resistor in the driving circuit of Figure 1(c) with *V_o_* as the output voltage. Given the input:

(2) $$V_{s2}=A_{S}\;\text{sin}\;(2\pi \;\mathrm{ft})$$

the output voltage *V_o_* can be expressed as:

(3) $$V_{o}=-A_{s}R_{g}\;\left(\frac{\text{sin}\;(2\pi \;\mathrm{ft})}{R_{s}}+2\pi \;{\mathrm{fC}}_{s}\;\text{cos}\;(2\pi \;\mathrm{ft})\right)$$

Then Equation (3) may be rewritten in terms of the phase shift, *θ*, and output amplitude, *A_o_*, as shown below:

(4) $$V_{o}=A_{o}\;\text{sin}\;(2\pi \;\mathrm{ft}+\theta )$$

Finally, combining Equations (3) and (4) yields:

(5) $$R_{s}=\frac{R_{g}A_{s}}{A_{o}\;\text{cos}\;(\theta )}$$

and

(6) $$C_{s}=\frac{A_{o}\;\sin(\theta )}{R_{g}A_{s}2\pi \;f}$$

Equation (6) was already used in [20,21] to demonstrate that the PFS exhibits a piezocapacitive response that is useful for reducing force-estimation errors.

## 3. Amplitude Nonlinearity of the PFS under DC Sourcing

Besides the already-identified frequency nonlinearity [21], the PFS exhibits amplitude nonlinearity. Initially, this study only looks at the nonlinearity for input voltages within the (−1 V, 1 V) range. An approach is then presented for higher input voltages. The analysis of amplitude nonlinearity is perforce split in two, because the sensor exhibits quasi-different responses, depending on the input voltage applied.

### 3.1. Modeling Amplitude Nonlinearity for Input Voltages below 1V

**Figure 2.**
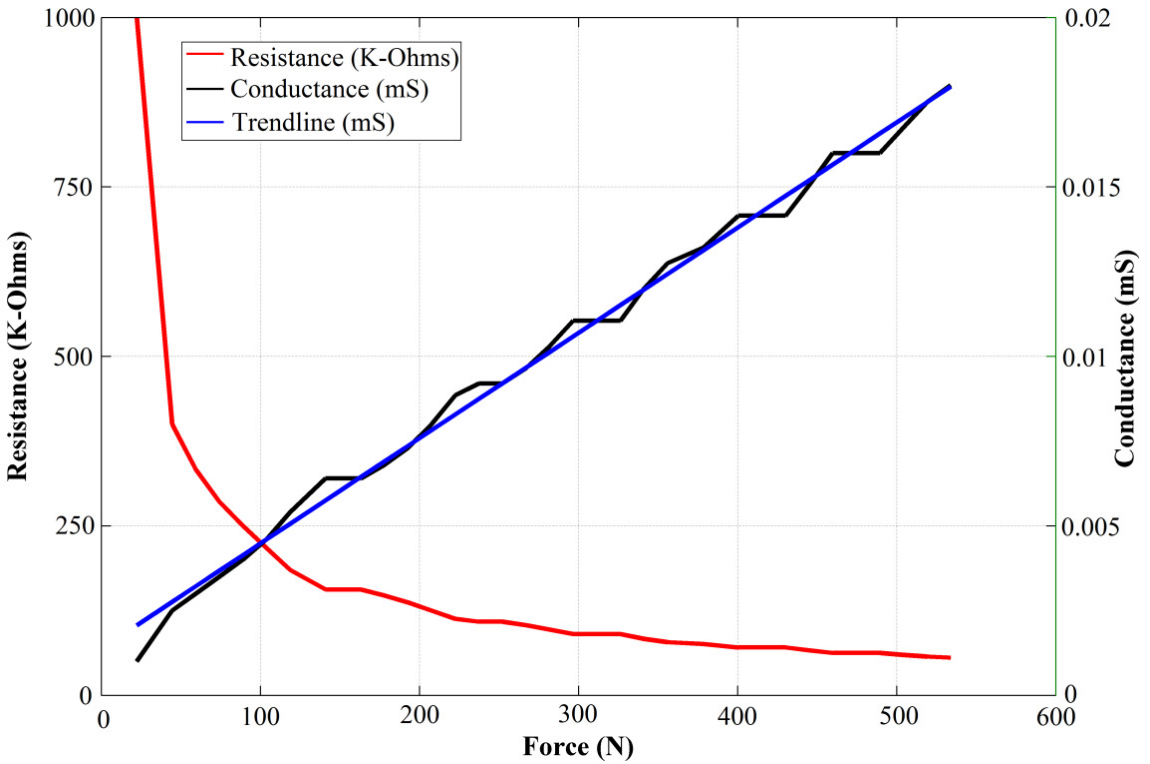
Typical variation of resistance and conductance for an A201-100 FlexiForce sensor (image taken from [25]; the image axis and legend were modified by the authors for better comprehension).

If the DC source, *V_s1_*, is chosen as the input of the driving circuit in Figure 1(c), a DC output voltage, *V_o_*, is obtained that changes linearly as the applied force on the sensor increases (see Figure 2) [25]. This response corresponds to the piezoresistive property of the sensor and it has been thoroughly described by the sensor manufacturer [24] and many research articles [4,7,8,17,20,22,26]. However, to the best of our knowledge, no information is available about how the output voltage changes for a fixed force when the DC voltage is varied.

**Figure 3.**
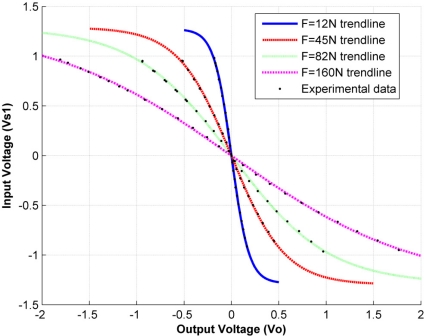
*V_s1_ vs. V_o_* for the PFS for driving voltages below 1 V and four different exerted forces of 12 N, 45 N, 82 N and 160 N. The trendline used for each individual fit was a hyperbolic tangent function [Equation (8)].

In order to study such behavior, the input voltage, *V_s1_*, is swept from −1 V up to 1 V, and the output voltage, *V_o_*, is plotted while the applied force remains constant. It is important to remark that these measurements are carried out by using a constant-puck area of 50.3 mm^2^, smaller than the sensor’s sensing area of 71.3 mm^2^, this ensures that every sensor is evenly loaded in each trial and avoids the pressure falling outside the sensing area of the sensor. Figure 3 shows the output voltage for randomly chosen forces of 12 N, 45 N, 82 N and 160 N. The best function for relating the input voltage, *V_s1_*, to the corresponding sensor response, *V_o_*, is found to be:

(7) $$V_{o}=-\frac{1}{q}\text{atanh}\;(\frac{V_{s1}}{k})$$

where *k* and *q* are constants. However, it is inadvisable to fit *k* and *q* in the form presented in Equation (7), because *V_o_* may yield complex values since the *atanh* domain is restricted to (−1, 1). Thus, for fitting purposes it is better to rewrite Equation (7) in terms of the *tanh* function as follows:

(8) $$V_{s1}=k\;\text{tanh}\;(-qV_{o})$$

The minus sign in Equations (7) and (8) comes from the negative gain in the inverting amplifier [see Figure 1(c)] used to drive the sensor. The axes in Figure 3 are intentionally switched to represent *V_o_* on the x-axis and *V_s1_* on the y-axis with the aim of fitting the data points with Equation (8) instead of Equation (7). The fitting process is highly fiable, with a coefficient of determination, *R^2^*, of at least *R^2^* = 0.9992 for every applied force and an average *R^2^* value of 0.9995.

**Figure 4.**
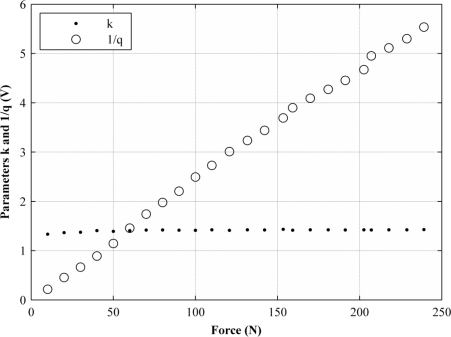
Graph representing the variation of sensor parameters *k* and *q* for different exerted forces within the range from 0 N to 250 N.

Parameters *k* and *q* were set to adjust independently for every applied force, however, the independent fitting processes returned values of *k* that remained almost constant, regardless of the exerted force, *F*, whereas *q* was shown to be hyperbolically dependent on the exerted force. In other words, *1/q* is a linear function of *F*. Figure 4 shows the variation of *k* and *1/q* for different applied forces within the 0 N–250 N range resulting from independent fitting processes.

In order to get a comprehensive view of sensor behavior, a relationship between Equation (8) and *F* must be found. For that purpose, the fact that the sensor exhibits piezoresistive behavior is useful, as thus its conductance, *1/R_s_*, may be modeled in terms of the applied force, *F*, as:

(9) $$1/R_{s}=\mathrm{m F}+b$$

Equation (9) is not explicitly stated in the PFS user manual [24], however, the sensor manufacturer does declare that a linear interpolation between the conductance values and the applied forces can be done. Also, it can be easily deduced from a look at the conductance curve in Figure 2 that Equation (9) is a valid fit for *1/R_s_*. Considering the inverting amplifier with feedback resistor *R_g_*, which is used to drive the PFS, it is possible to link Equation (9) with the amplifier characteristic equation:

(10) $$\frac{V_{o}}{V_{s1}}=-\frac{R_{g}}{R_{s}}$$

to obtain:

(11) $$\frac{V_{o}}{V_{s1}}=-R_{g}\;(\mathrm{mF}+b)$$

We should clarify that Equation (11) is not explicitly stated in the PFS user manual [24], and only Equation (10) is given in [24], but the manufacturer suggests that the sensor sensitivity, *m*, can be changed by either replacing the feedback resistor, *R_g_*, or changing the driving voltage, *V_s1_*.

With the aim of demonstrating that Equation (11) is an approximate expression for fitting the data points from Figure 3, the *1/q* curve from Figure 4 is taken and represented as linearly dependent on the applied force:

(12) $$1/q=m^{\prime }F+b^{\prime }$$

Note that the *1/q* curve in Figure 4 is analogous to the conductance curve of Figure 2. Equation (12) can be substituted into Equation (7), yielding:

(13) $$V_{o}=-(m^{\prime }F+b^{\prime })\;\text{atanh}\;\left(\frac{V_{s1}}{k}\right)$$

Nevertheless, Equation (13) cannot be stated the same way as Equation (11), because the input voltage, *V_s1_*, is part of the *atanh* argument, but if only the first term of the *atanh*-Taylor series is taken, the following approximate expression is obtained:

(14) $$\frac{V_{o}}{V_{s1}}=\frac{-1}{k}\;(m^{\prime }F+b^{\prime })$$

Equation (14) is an approximate expression for modeling sensor response, and so is Equation (11). The *1/k* factor in Equation (14) is analogous to *R_g_* in Equation (11), in the same way that *m* is analogous to *m′*, *b* to *b′* and *1/R_s_* to *1/q*. Equations (11) and (14) are valid if and only if the input voltage remains constant during the measurement process; this condition matches the driving conditions recommended by the manufacturer [24].

### 3.2. Effect of the Feedback Resistor in Sensor Response

The effect of the feedback resistor can be deduced from the fact that *R_g_* only changes the feedback gain of the amplifier, without affecting sensor current. Thus, changing *R_g_* will produce directly proportional changes in the output voltage, and Equation (13) can be rewritten as:

(15) $$V_{o}=-\left(\frac{R_{g}}{R_{\mathrm{ref}}}\right)\;(m^{\prime }F+b^{\prime })\;\text{atanh}\;\left(\frac{V_{s1}}{k}\right)$$

Henceforth Equation (15) is referred to as “the general-sensor model” under DC sourcing, with the restriction |*V_s1_*| < *1 V*, where *R_ref_* is the feedback resistor used during the characterization to obtain the values of *m′*, *b′* and *k*. In case the feedback resistor is changed after the characterization process, the output voltage is multiplied by the ratio *R_g_/R_ref_* where *R_g_* is the new feedback resistor. Replacing the feedback resistor produces a directly proportional change in the output voltage, because the amplifier is inherently linear, whereas sensor resistance is not. In fact, linking Equations (7), (10) and (12) produces an expression that shows the nonlinear behavior of sensor conductance in response to changes in the input voltage:

(16) $$\frac{1}{R_{s}}=(m^{\prime }F+b^{\prime })\frac{\text{atanh}\;(V_{s1}/k)}{V_{s1}\;R_{g}}$$

However, it must be noted from Equation (16) that sensor conductance is always linear to force changes.

### 3.3. Generalized Method for Obtaining a Specific Sensitivity in Sensor Response

Since a general-sensor model was deduced in Equation (15), a generalized method can be presented for obtaining a specific sensitivity in the sensor. The tests discussed in this section were done using four A201-100 sensors to provide more representative results. Each PFS exhibits a considerably different sensitivity. This condition prevents the sensors from having plug-and-play capability, because a characterization must be run before using a sensor. Moreover, for characterizations run at a given input voltage, it has been impossible until now to determine the new values of *m* and *b* under the new input voltage condition, because the amplitude nonlinearity was not accounted for. Nevertheless, Equation (15) accounts for changes in both the input voltage, *V_s1_*, and the feedback resistor, *R_g_*. This implies that if *m′*, *b′*, *k*, and *R_ref_* are given, it is possible to either determine sensor sensitivity for any input voltage and feedback resistor or design a specific driving circuit ,*V_s1_* and *R_g_*, with the aim of matching a target sensitivity.

Note that the *k* factor in Equation (15) contains information about sensor sensitivity for any input voltage. Unfortunately, the *k* factor is different for each sensor, just as sensitivity is too. The range of variation of the *k* factor for the four sensors under study was from 1.2 V to 1.5 V, with an average value of 1.41 V. If *k* is not given, the only way to change sensor sensitivity is to replace the feedback resistor, *R_g_*; but, in certain applications where several sensors are being used [1,16,21,23], it is more convenient to change the driving voltage instead of changing individual feedback resistors. In addition, the feedback resistor is increased despite an increase in the noise level, so it is better to match a desired sensitivity by making a trade-off between *V_s1_* and *R_g_*.

With the aim of proposing a method for obtaining a specific PFS sensitivity, it is necessary to fit the general-sensor model in Equation (15) to the experimental data. However, the feedback resistor must not play any role in the fitting process, because *R_g_* only affects the closed loop gain of the amplifier, not the sensor response itself. Thus, it is better to use Equation (13) for the fit instead of Equation (15). In order to avoid complex numbers resulting from Equation (13) during the fit, Equation (13) is rewritten in terms of the *tanh* function as shown below:

(17) $$V_{s1}=-k\;\text{tanh}\;\left(\frac{V_{o}}{m^{\prime }F+b^{\prime }}\right)$$

Note that Equation (17) implies a three-dimensional fit in terms of the variables *F*, *V_o_* and *V_s1_*, with coefficients *m′*, *b′* and *k* to be determined.

In practice, the experimental data were gathered in the same way as described in Section 3.1. Nevertheless, the fit here described embraces all variables and coefficients at once (*F*, *V_o_*, *V_s1_*, *m′*, *b′* and *k*), whereas the fitting process in Section 3.1 was split into two independent threads. This is an important difference, because the fit here tries to minimize the overall error, while the process in Section 3.1 independently minimizes the error for every exerted force.

**Figure 5.**
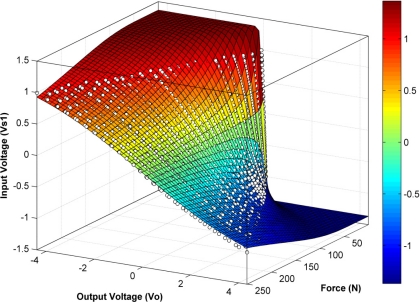
Surface resulting from the three dimensional fit of the PFS-general model to the experimental data points shown as empty circles in the plot.

Figure 5 shows the experimental data points and the surface produced by the three-dimensional fit in Equation (17). For this case, the values of *R^2^* were in general lower than for the fits given in Section 3.1. The minimum value of *R^2^* for the four sensors under study was *R^2^* = 0.989, with an average value of *R^2^* = 0.991; this is comprehensible, because all forces and voltages were embraced in a single fit, and thus a single *k* value was returned.

Once the fit is done, it is possible to assemble the general-sensor equation by substituting into Equation (15) the coefficient values returned from the fit (*m′*, *b′* and *k*) and the value of the feedback resistor used during the characterization process, which matches *R_ref_* in Equation (15). If *R_g_* or *V_s1_* are changed at any time, it is possible to recalculate *m* and *b* from Equation (15); or, if the feedback resistor, *R_g_*, is fixed by the application circuit, the target sensitivity, *m_t_*, can be obtained by choosing the appropriate *V_s1_* from Equation (15) as shown below:

(18) $$V_{s1}=k\;\text{tanh}\;\left(\frac{m_{t}}{m^{\prime }}\frac{R_{\mathrm{ref}}}{R_{g}}\right)$$

**Figure 6.**
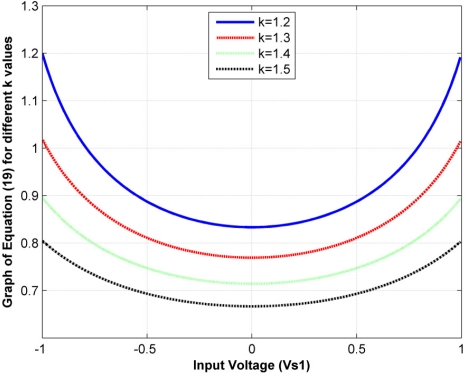
Graph of the Equation (19) for different *k* values. Note that as *k* increases the lines flatten.

However, Equation (18) is valid if and only if the predicted input voltage is lower than 1 V, because only the amplitude nonlinearity for |*V_s1_*| < *1 V* has been studied. Note that the *k* factor may be taken as a mean of amplitude nonlinearity. To confirm this, Figure 6 plots the following function, which is drawn from the sensor-conductance expression, Equation (16):

(19) $$G(V_{s1})=\frac{\text{atanh}\;(V_{s1}/k)}{V_{s1}\;R_{g}}$$

Equation (19) is a measure of how the sensor conductance is affected by changes in the input voltage. The higher *k* is for a given sensor, the flatter the lines in Figure 6. This can be understood as a less-noticeable amplitude nonlinearity in the sensor.

### 3.4. An Approach to Modeling the Amplitude Nonlinearity for Input Voltages above 1 V

**Figure 7.**
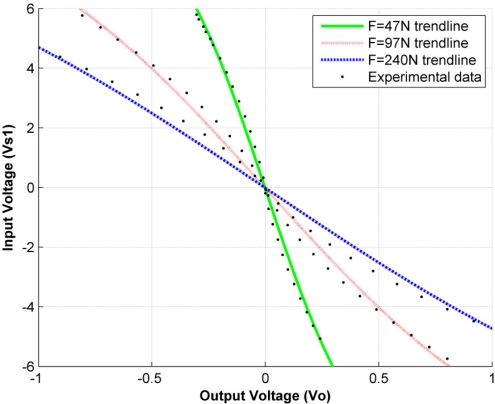
Graph representing the relation of *V_s1_ vs. V_o_* for input amplitudes within the range (−6 V, 6 V) and forces of 47 N, 97 N and 240 N.

If the PFS is sourced with voltages above 1V, the fitting curve in Equation (8) and the general-sensor model in Equation (15) provide an unsatisfactory fit according to our criteria. Figure 7 shows the output and input voltage from data taken experimentally under three different forces (47 N, 97 N and 240 N) for driving voltages within the range (−6 V, 6 V) with Equation (8) as the trendline. Note that the experimental data points move away from the trendline, especially in the middle-range voltages, |*V_s1_*| < *3 V*.

Despite the fact that the coefficient of determination remained high for all sensors under study (exhibiting an average value of *R^2^* = 0.991), it is clear that Equations (8) and (15) require some changes in order to provide a better fit. So far we have not found a suitable and simple curve for modeling sensor behavior under such driving conditions.

## 4. Effect of Amplitude Nonlinearity under AC Sourcing for the Sensor

It has been demonstrated that sensor conductance is not constant if there are changes in the input voltage. By relating Equation (1) with the conductance model, Equation (16), the following expression is obtained, which is useful for studying the effect of amplitude nonlinearity under AC sourcing:

(20) $$-V_{o}=(m^{\prime }F+b^{\prime })\;\text{atanh}\;\left(\frac{V_{s2}}{k}\right)+R_{g}C_{s}\frac{d}{\mathrm{dt}}(V_{s2})$$

where |*V_s2_*| < 1*V*, ∀*t* in order to meet the general-sensor model, Equation (15).

Under AC sourcing, the nonlinear term, Equation (19), extracted from Equation (16) causes a modulation effect in sensor conductance which was not accounted for in our previous work (summarized in Section 2). It is evident that solving Equation (20) for a sine-wave input yields a set of equations different from Equations (3–6). But, if the input amplitude of the AC signal is low enough, sensor conductance can be taken as approximately constant and consequently, the expressions in Section 2 may be taken as valid.

Specifically, the frequency analyses carried out in [20,21] were done under a sine-wave input with *A_s_* = *0.5 V*, and therefore the results reported by those sources can be trusted. Note from Figure 3 that for |*V_s1_*| < *0.5 V*, sensor conductance can be taken approximately as constant regardless of changes in *V_s1_*.

In contrast, the tests carried out in [20,21] for estimating the effect of capacitance readings in force-estimation errors are in principle questionable, because the input signal used for those tests was a sine wave with *A_s_* = 3 V. Nevertheless, considering that [20,21] reported an effective reduction in force-estimation errors, we hypothesize that amplitude nonlinearity does not significantly affect capacitance readings. Unfortunately, we are unable to estimate the underlying error stemming from the capacitance readings reported in [20,21], whether the input sine wave has an amplitude of 0.5 V or 3 V. Where the input sine wave has an amplitude of 0.5 V, Equation (20) must be solved analytically in terms of *θ* and *A_o_*, which is a challenging task beyond the scope of this article; and where the input sine wave is 3 V, a comprehensive model of the amplitude nonlinearity must be developed for input voltages above 1V, and then such model must be included in the differential Equation (1).

Neither procedure is addressed in this paper, because a method is proposed in Section 5 for bypassing the amplitude nonlinearity under AC operation, regardless of whether the input voltage is lower or higher than 1 V.

Hyperbolic-tangent nonlinearity was hard to detect in our previous work [20,21], because it produces neither saturation nor exponential growth in the output voltage. An additional circumstance prevents the detection of amplitude nonlinearity; to show this, *V_s2_* was replaced in Equation (20) with a sine function, and Equation (12) was used to state Equation (20) in terms of *q* as shown below:

(21) $$V_{o}=-\left[\frac{1}{q}\text{atanh}\;\left(\frac{A_{s}}{k}\;\text{sin}\;(2\pi \;\mathrm{ft})\right)+2\pi \;{\mathrm{fC}}_{s}R_{g}A_{s}\;\text{cos}(2\pi \;\mathrm{ft})\right]$$

A close look at Equation (21) reveals that the output voltage, *V_o_*, is the sum of the nonlinear term stemming from sensor conductance and the term stemming from sensor capacitance. It is demonstrated in Section 6 that sensor capacitance is constant, regardless of changes in the input voltage, and therefore the resulting *V_o_* in Equation (21) is the sum of a linear and a nonlinear term. From the time-domain viewpoint, given an input sine wave, the resulting output, Equation (21), looks rather sinusoidal, because the nonlinear response is diminished by the linear response. From the frequency-domain viewpoint, the output voltage, Equation (21), is slightly distorted because of the odd harmonics coming from the high-order terms of the *atanh*-Taylor series.

## 5. Bypassing Amplitude Nonlinearity for Estimating Sensor Capacitance

Factors *q* and *k* can be easily determined if the sensor is sourced with a DC signal; there is therefore no interest in estimating *q* and *k* using AC sourcing. Note in Equation (21) that sensor capacitance, *C_s_*, is multiplied only by the term with the cosine function, so if that term can somehow be isolated, it may become possible to read sensor capacitance.

Multiplying *V_o_* from Equation (21) by:

(22) $$V_{x}=A_{x}\;\text{cos}\;(2\pi \;\mathrm{ft})$$

and low-pass filtering (LPF) yields this product:

(23) $$\mathrm{LPF}\;[V_{o}V_{x}]=-\mathrm{LPF}\;[A_{x}\;\text{cos}\;(2\pi \;\mathrm{ft})\frac{1}{q}\text{atanh}\;\left(\frac{A_{s}}{k}\;\text{sin}\;(2\pi \;\mathrm{ft})\right)+A_{x}\;\text{cos}\;(2\pi \;\mathrm{ft})\;2\pi \;{\mathrm{fC}}_{s}R_{g}A_{s}\;\text{cos}\;(2\pi \;\mathrm{ft})]$$

Considering that the composition of two odd functions is an odd function, the first term of Equation (21) is consequently odd (as the *atanh* and *sin* functions are both odd). Multiplying such a term by an even function, Equation (22), results in an odd function with frequency components at 4*πnf*, ∀*n* ≥ 1 ∈ *N*

The harmonics come from the decomposition of the *atanh* function in its Taylor series. Thus, the first term of Equation (23) can be expressed as:

(24) $$\begin{array}{l} \frac{1}{q}\;\text{atanh}\;\left(\frac{A_{s}}{k}\;\text{sin}\;(2\pi \;\mathrm{ft})\right)A_{x}\;\text{cos}\;(2\pi \;\mathrm{ft})=\frac{A_{x}\;\text{cos}(2\pi \;\mathrm{ft})}{q}[\frac{A_{s}}{k}\;\text{sin}(2\pi \;\mathrm{ft})+\\ -\frac{1}{3}{\left(\frac{A_{s}}{k}\;\text{sin}\;(2\pi \;\mathrm{ft})\right)}^{3}+\frac{2}{15}{\left(\frac{A_{s}}{k}\;\text{sin}\;(2\pi \;\mathrm{ft})\right)}^{5}+\ldots ] \end{array}$$

Note that Equation (24) does not have a DC component, and thus the low-pass filter removes the entire signal. On the other hand, the second term of Equation (23) is an even function and may be rewritten as:

(25) $$2\pi \;{\mathrm{fC}}_{s}R_{g}A_{s}\;\text{cos}\;(2\pi \;\mathrm{ft})\;A_{x}\;\text{cos}\;(2\pi \;\mathrm{ft})=\pi \;{\mathrm{fA}}_{s}A_{x}R_{g}C_{s}\;(1+\text{cos}(4\pi \;\mathrm{ft}))$$

Equation (25) has a nonzero mean value and a term with a frequency component at *4πf*, which is removed by the LPF, and thus it can be simplified to:

(26) $$\mathrm{LPF}\;[V_{o}V_{x}]=-\pi \;{\mathrm{fA}}_{s}A_{x}R_{g}C_{s}$$

**Figure 8.**
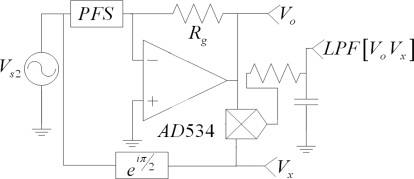
General diagram for measuring *C_s_* by means of removing the conductance term from the output voltage by performing the operation *LPF[V_o_V_x_]*.

The above expression is useful for reading sensor capacitance, because the other factors in Equation (26) are all constants. The mathematical function *LPF[V_o_V_x_]* can be obtained by using a four-quadrant multiplier, such as the AD534, and an RC-series circuit in the low-pass configuration. Figure 8 summarizes the described process for measuring *C_s_*.

Equation (26) is inherently different from Equation (6), because sensor conductance is not present in the former, while in the latter it is, albeit implicitly, due to the ratio *A_o_/A_s_* in Equation (6). This is an important difference, because the process depicted here removes the contribution of sensor conductance from the output signal. So, regardless of what the conductance model is, Equation (26) remains unchangeable. We are unable to provide a theoretical demonstration of this statement until a model for *1/R_s_* is developed for sourcing voltages above 1 V; however, in the next section we present experimental results that support this hypothesis.

We would like to stress that different methods for measuring capacitance were evaluated [27–30], but the fact that a variable resistor, *R_s_*, is placed across the capacitance, *C_s_*, makes reading difficult. The method described above was inspired by the synchronous demodulation of RF signals [31]. Note that *C_s_* can be considered as the message in Equation (25), the term 2*πfR_g_A_s_*cos(2*πft*) acts like the carrier signal and *A_x_*cos(2*πft*) as the demodulating signal.

## 6. Experimental Results of Conductance and Capacitance Readings for Different Input Voltages

With the aim of demonstrating the statements presented herein, we carried out a series of capacitance and conductance measurements on four PFSs under different sourcing and force conditions. The tests are classified below according to sourcing type.

### 6.1. DC Sourcing

Forces were applied within the range of 0 N to 250 N under seven sourcing conditions, starting at 0.4 V, with increments of 0.1 V, up to 1 V. For every supply voltage, the sensor sensitivity, *m*, and the y-intercept, *b*, were found according to the classical method proposed by the manufacturer [24]. These values of *m*, *b* are used as references for calculating subsequent errors in *m*, *b* for the different methods presented in this article.

Sensors were then characterized in terms of *m′*, *b′* and *k* as described in Section 3.3. Doing so enabled Equation (15) to be assembled and *m*, *b* to be estimated for the aforementioned discrete DC voltages.

With the aim of demonstrating the typical error resulting from the assumption of linear-sensor response, the values of *m*, *b* measured at 1 V and Equation (11) were used to estimate *m*, *b* for the remaining six voltages (0.4 V to 0.9 V). That is, if the *m* value measured at 1 V is 20 mV/N, use of Equation (11) shows that the estimated value of *m* at 0.4 V is equal to 8 mV/N; the same calculation applies to the y-intercept coefficient, *b*. Table 1 compares the errors of *m* and *b* predicted from the linear, Equation (11), and the nonlinear model, Equation (15).

**Figure 9.**
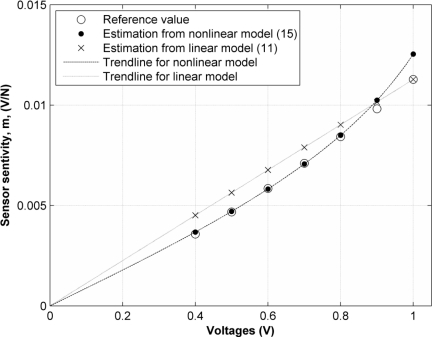
Sensor Sensitivity, *m*, for different input voltages. The prediction from the linear Equation (11) and the nonlinear Equation (15) models are compared to the reference sensitivity measured at different input voltages.

Two facts may be drawn from Table 1. First, the errors in the *m* values predicted by Equation (15) are rather small for all sensors. This means that the nonlinear model presented in this paper produces matches in predicting the sensor sensitivity for any input voltage under 1 V. In fact, the average error in *m* for all sensors and all voltages is only 2.04%. Second, the assumption of linear response in sensor conductance produces a reasonably greater error when predicting *m via* Equation (11); an average error of 11.35% was obtained, which is almost six times the error resulting from Equation (15). Figure 9 shows the values of *m* for sensor number four, under all the discrete applied voltages. Also shown are the trendlines arising from the linear Equation (11) and the nonlinear model, Equation (15).

Second, the average error for all sensors resulting from the estimation of the y-intercept coefficient, *b*, was quite high, 10.27% and 14.5%, for the two models. Despite this fact, we declare that Equation (15) can effectively model sensor behavior, because through our various optimization processes we have effectively reduced the estimation error of *b* by discarding the highly noisy data obtained under forces lower than 40 N. Such data are noisy because of the low amplitude of *V_o_*, resulting from the low-force condition. If the data taken under forces lower than 40 N are discarded, the average error for the y-intercept estimation is reduced to 6.35%.

### 6.2. AC Sourcing

**Figure 10.**
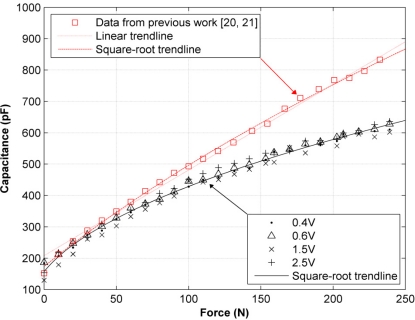
Sensor capacitance, *C_s_*, measured using two different methods under different sourcing voltages.

Input amplitudes are not restricted to be under 1 V for the AC sourcing experiments, because the intention is to subject the feasibility of the measuring scheme in Figure 8 to a thorough test. Figure 10 shows two different sets of capacitance values, *C_s_*, for the same sensor, taken under sinusoidal excitation at a fixed frequency of 4 KHz. The black values were taken according to the measuring scheme in Figure 8, whereas the red data points were taken from our previous work [20,21], when the amplitude nonlinearity was unknown and the equations in Section 2 were used to estimate *C_s_*. This set of data was taken at a fixed input amplitude of 3 V.

Considering:

1. That the black data points in Figure 10 are rather close to each other for the different driving voltages, and
2. That the feasibility of the measuring scheme in Figure 8 was theoretically demonstrated in Section 5

it may be concluded that sensor capacitance is constant, regardless of changes in the driving voltage, with the restriction of |*V_s1_*| < 1 V. A look at the capacitance values for input voltages of 1.5 V and 2.5 V (see Figure 10) shows that it is reasonable to suggest that *C_s_* remains constant for *V_s1_* above 1 V, but this cannot be definitely stated until a model for sensor conductance is developed for *V_s1_* above 1 V, because the compatibility of such model with the measuring scheme in Figure 8 must be ascertained first.

An attempt was made to fit a linear trendline to the black data points in Figure 10. This is a logical approach, considering that our previous work reported a linear variation of *C_s_* in response to force changes [20,21]. However, the residuals resulting from the fitting process suggested that a linear fit was unsuitable. Thus, different trendline forms were tried. The best, but also simplest, trendline that could model the variation of *C_s_* was a square-root model dependant on the applied force, *F*:

(27) $$C_{s}=C_{o}+a\sqrt{F+b}$$

where *a*, *b* and *C_o_* are constants estimated from the fit.

On the other hand, the red data points in Figure 10, taken from our previous work [20,21], were fitted with a line, and an acceptable coefficient of determination was obtained, *R^2^* = 0.9942. The same data points were fitted with Equation (27), and the value of *R^2^* was even better, *R^2^* = 0.9987; this means that in principle either trendline is valid for fitting the data.

From Figure 10, it is clear that one set of data points is incorrect, because it is impossible that different values of capacitance are measured for the same sensor, under the same applied forces and the same mechanical layout. Considering that the red data points were taken under the erroneous supposition of linear conductance, it is logical to suggest that it was the assumption that yielded the incorrect values of capacitance; but, as stated in Sections 4 and 5, there are a few steps remaining to be accomplished before this hypothesis can be demonstrated.

In other words, we have theoretically and experimentally demonstrated that the black data points from Figure 10 are correct at least for |*V_s1_*| < 1 V, but we are unable to theoretically demonstrate that the error basis from the red data points is due to the assumption of linear sensor conductance, despite some experimental results supporting this hypothesis. For instance, a look at the measured capacitance under low applied forces in Figure 10 shows that both methods estimate the same *C_s_*. This may be understood if one recalls the piezoresistive behavior of the sensor; so, under low applied forces and AC excitation, sensor resistance can be taken as virtually infinite (See Figure 2), and its response is dominated only by capacitive factors. Under these circumstances, the resistive term in Equation (1) may be discarded, and the measured capacitance will be the same, regardless of the conductance model or the method employed for measuring *C_s_*. It is only when the exerted force is increased that the aforementioned conditions start to play an important role in the estimation of sensor capacitance, and consequently, as *F* increases, the two sets of data points gradually diverge.

## 7. Conclusions and Future Work

For sourcing voltages below 1 V, a comprehensive model for the FlexiForce model A201-100 piezoresistive device has been developed and tested. A nonlinear response has been identified in sensor conductance corresponding to a hyperbolic tangent function.

A general method for obtaining a specific sensitivity, *m*, in sensor response has been presented and experimentally tested on four FlexiForce sensors; such method produced an average error of 2.04% when estimating *m*. Whereas the y-intercept, *b*, estimation yielded a considerable greater error of 10.27%; however such error can be diminished by discarding the noisy data resulting from the highly noisy data obtained under forces lower than 40 N. The method presented in this article has considerably improved the plug-and-play capability of the piezoresistive device.

The authors have theoretically and experimentally demonstrated that sensor capacitance is constant regardless of changes in the input voltage whenever the driving voltage is held below 1 V. The feasibility of a measuring scheme for bypassing conductance nonlinearity has also been presented and tested.

Conversely, the experimental results support the hypothesis of constant capacitance for input voltages above 1 V. Nonetheless, it is necessary to develop a theoretical model for sensor conductance for sourcing voltages above 1V before declaring that sensor capacitance remains unchangeable at such sourcing voltages. Future work will mainly focus on adding the puck area as a variable in the general-sensor model. This would allow the final user to assess how the sensor sensitivity is affected when the puck area is changed.

## Figures and Tables

**Table 1.** Comparison table of the average errors resulting from the estimation of *m, b* by means of a linear Equation (11) and a nonlinear Equation (15) under input voltages within the range (0.4 V, 0.9 V).

**Sensor number**	**1**	**2**	**3**	**4**	**Average error for all sensors**
Average error (%) in *m* resulting from (15)	2.79	1.07	2.67	1.62	2.04
Average error (%) in *m* resulting from (11)	10.3	10.3	10.8	14	11.35
Average error (%) in *b* resulting from (15)	9.46	12.5	10.9	8.2	10.27
Average error (%) in *b* resulting from (11)	13.5	20.3	13.3	10.9	14.5
